# Whipworms in humans and pigs: origins and demography

**DOI:** 10.1186/s13071-016-1325-8

**Published:** 2016-01-22

**Authors:** Mohamed B. F. Hawash, Martha Betson, Azmi Al-Jubury, Jennifer Ketzis, Arve LeeWillingham, Mads F. Bertelsen, Philip J. Cooper, D. Tim J. Littlewood, Xing-Quan Zhu, Peter Nejsum

**Affiliations:** Department of Veterinary Disease Biology, Faculty of Health Sciences, Copenhagen University, Dyrlaegevej 100, DK-1870 Frederiksberg C, Copenhagen Denmark; Zoology Department, Faculty of Science, Cairo University, Giza, Egypt; Department of Production and Population Health, Royal Veterinary College, Hatfield, Hertfordshire, UK; School of Veterinary Medicine, University of Surrey, Guildford, Surrey, UK; Ross University School of Veterinary Medicine, West Indies, Basseterre, St Kitts and Nevis; Centre for Zoo and Wild Animal Health, Copenhagen Zoo, Frederiksberg, Copenhagen Denmark; Institute of Infection and Immunity, St George’s University of London, London, UK; Department of Life Sciences, Natural History Museum, London, UK; Lanzhou Veterinary Research Institute, State Key Laboratory of Veterinary Etiological Biology, Key Laboratory of Veterinary Parasitology of Gansu Province, Chinese Academy of Agricultural Sciences, Lanzhou, Gansu Province 730046 P R China

**Keywords:** Whipworms, *Trichuris*, Humans, Pigs, Demographic history, Evolution

## Abstract

**Background:**

*Trichuris suis* and *T. trichiura* are two different whipworm species that infect pigs and humans, respectively. *T. suis* is found in pigs worldwide while *T. trichiura* is responsible for nearly 460 million infections in people, mainly in areas of poor sanitation in tropical and subtropical areas. The evolutionary relationship and the historical factors responsible for this worldwide distribution are poorly understood. In this study, we aimed to reconstruct the demographic history of *Trichuris* in humans and pigs, the evolutionary origin of *Trichuris* in these hosts and factors responsible for parasite dispersal globally.

**Methods:**

Parts of the mitochondrial *nad*1 and *rrn*L genes were sequenced followed by population genetic and phylogenetic analyses. Populations of *Trichuris* examined were recovered from humans (*n* = 31), pigs (*n* = 58) and non-human primates (*n* = 49) in different countries on different continents, namely Denmark, USA, Uganda, Ecuador, China and St. Kitts (Caribbean). Additional sequences available from GenBank were incorporated into the analyses.

**Results:**

We found no differentiation between human-derived *Trichuris* in Uganda and the majority of the *Trichuris* samples from non-human primates suggesting a common African origin of the parasite, which then was transmitted to Asia and further to South America. On the other hand, there was no differentiation between pig-derived *Trichuris* from Europe and the New World suggesting dispersal relates to human activities by transporting pigs and their parasites through colonisation and trade. Evidence for recent pig transport from China to Ecuador and from Europe to Uganda was also observed from their parasites. In contrast, there was high genetic differentiation between the pig *Trichuris* in Denmark and China in concordance with the host genetics.

**Conclusions:**

We found evidence for an African origin of *T. trichiura* which were then transmitted with human ancestors to Asia and further to South America. A host shift to pigs may have occurred in Asia from where *T. suis* seems to have been transmitted globally by a combination of natural host dispersal and anthropogenic factors.

**Electronic supplementary material:**

The online version of this article (doi:10.1186/s13071-016-1325-8) contains supplementary material, which is available to authorized users.

## Background

A range of mammalian hosts is infected with parasitic whipworms belonging to the genus *Trichuris*. Around 460 million humans are infected with *T. trichiura* mainly in developing countries in South and South-East Asia, Sub-Saharan Africa and Latin America [1]. The prevalence of whipworms in non-human primates is generally high and despite the fact that the taxonomic status is unsettled, they are historically designated as *T. trichuris* [2]. *T. suis* infection in pigs is globally widespread with the highest prevalence in young pigs reared in outdoor production systems [3, 4]. A number of studies have investigated the genetic relationship among pig and primate derived *Trichuris* but their evolutionary relationship is still poorly understood and in particular the anthropogenic and environmental factors responsible for their global distribution.

Population genetic tools provide a valuable opportunity to investigate the epidemiological history and transmission of parasites and have been adopted to study many parasitic nematodes [5, 6]. For instance, population genetic approaches were applied recently to investigate the pattern of transmission of *Ascaris suum* and *A. lumbricoides* between pigs and humans across the globe [7]. Moreover, population genetics can be used to reconstruct the epidemiological and demographic history of micro- and macro- parasites through coalescent analysis coupled with Bayesian approaches [5]. Reconstruction of the epidemiological and demographic history gives us a window into the past to see which factors facilitated spread or the introduction of the parasites to new regions [5]. For instance, the demographic history of *Wuchereriabancrofti* suggests that this parasite was introduced to India 60,000-70,000 years ago through human migration out of Africa. In contrast, the introduction of *W. bancrofti* to Papua New Guinea cannot be explained by ancient human migration but must have been introduced through more recent human migration [8].

During the evolution of genus *Homo* nearly 4 million years ago, there has been continuous contact with many parasites. Those infecting humans today may have different evolutionary origin and can be categorized into two groups: 1) Parasites transmitted to humans through primate common ancestors and are referred to as “heirloom” and 2) parasites acquired more recently through contact with animals (e.g. during animal domestication in the Neolithic period roughly 10,000 years ago) and are referred to as “souvenirs” [9]. The human whipworm is generally considered heirloom as it is found in the African non-human primates, and parasite eggs were found in human coprolites in archaeological sites before animal domestication and in the New World before the Columbian colonization [10–12]. However, there is no rigorous genetic evidence for this assumption especially with recent studies suggesting that several *Trichuris* species can be found in non-human primates such as the *Trichuris* sp. in Francois leaf monkey in China and *Trichuris* spp. infecting a number of African non-human primates in Uganda [13, 14]. Hence, the genetic and evolutionary relationship between *Trichuris* from humans and non-human primates is poorly understood.

Different molecular markers have been used to resolve the genetic and evolutionary relationship between *Trichuris* spp. isolates from different host speices. For instance, several studies have used the nuclear ribosomal internal transcribed spacers (ITS) to investigate the genetic relationship among *Trichuris* from humans, non-human primates and pigs [13, 15, 16] whereas other studies relied on small subunit ribosomal DNA (18S) and the mitochondrial *cox*1 gene [17, 18]. Full mitochondrial genome analysis has been used in some studies to investigate the phylogenetic relationship of *Trichuris* spp. but includes only a limited number of worm samples [14, 19, 20].

Herein, we investigated the genetic and evolutionary relationships between populations of *Trichuris* parasites derived from humans, non-human primates and pigs from different continents. The mitochondrial *nad*1 and *rrn*L genes of 138 worms were sequenced and coalescent simulations applied to infer the demographic history of the different human and pig *Trichuris* populations and their evolutionary origin.

## Methods

### Ethics statement

The human *Trichuris* from Uganda and Ecuador were recovered from the faeces of children and adults after anthelmintic treatment. Permission was obtained from the Ministry of Health and the National Council of Science and Technology in Uganda and by the Ethical Committees of Liverpool School of Tropical Medicine and Pontificia Universidad Catolica del Ecuador in Ecuador. The Danish Central Medical Ethics Committee approved the study. Informed written consent was obtained from all participants or their guardians (for children) after being informed about the study in both English/Spanish and the local language.

Worms from baboons at Southwest National Primate Research Center, Texas, USA, Copenhagen Zoo, Denmark, Knuthenborg Park, Denmark and African Green Monkeys at Saint Kitts were recovered during post mortem examination which is performed routinely at these places on culled animals.

*T. suis* was obtained from experimentally infected pigs in Denmark and US. The Animal Experiments Inspectorate, Ministry of Justice, DK approved the animal study protocol and was carried out according to their guidelines (Licence no. 2005/561-1060). In US the animals were handled and managed according to a protocol (Protocol #07-011) specifically approved by the USDA-ARS Beltsville Area Animal Care and Use Committee and followed the Institutional Animal Care and Use Committees (IACUC) guidelines. In Uganda *T. suis* was obtained from naturally infected pigs raised on private farms, slaughtered and used for local consumption. Permission to recover worms was obtained from the owners. In Ecuador, worms were collected after anthelmintic treatment of a naturally infected pig raised on a private farm. Written consent to recover worms was obtained from the owner. *T. suis* were obtained from natural infected pigs in China when slaughtered at local abattoirs. Collection was approved by the Animal Ethics Committee of the Lanzhou Veterinary Research Institute, Chinese Academy of Agricultural Sciences.

### Parasite isolates, DNA extraction and typing of the worms

A total of 138 worms were collected from humans, pigs and non-human primates from different regions (Table 1). All worms were rinsed with tap water and stored in 70 % ethanol at 5 °C until DNA extraction. The MasterPure DNA Purifications Kit (Epicentre Biotechnologies) was used to extract DNA according to manufacturer’s protocol after homogenization in 300 μl of lysis solution and overnight incubation at 56 °C except for the populations from Ecuador for which the DNA was extracted previously [21]. Worms were initially typed to confirm worm species by Polymerase Chain Reaction-Restriction Fragment Length Polymorphism (PCR-RFLP) on internal transcribed spacer-2 (ITS-2) following the protocol described by [22]. A negative water control was included in all runs. PCR products and RFLP fragments were stained by GelRed (Biotium) and visualized under UV light in 1.5 % agarose gel. All *Trichuris* from baboons and humans showed banding pattern characteristic of *Trichuris* from primates, while all *T. suis* worms showed the banding pattern characteristic of *T. suis*.

**Table 1:** A summary of the number of *Trichuris* isolates, the host from which samples were recovered, the country of origin and sampling location(s)

Host (host numbers)	Country (number of samples)	Sampled localities in each country (number of samples)	Reference
Domesticated pigs, *Sus domesticus* (10)	Uganda (18)	Villages ranged 30 Km apart in south west Kabale district (18)	[22]
Domesticated pigs, *Sus domesticus* (5)	China (14)	Guangdong Province (3), Fujian Province (3), ChongqingMunicipality (4), Hunan Province (4)	This study
Domesticated pigs, *Sus domesticus* (2)	Denmark (10)	Experimentally infected pigs with local strains of the parasite (10)	[22]
Domesticated pigs, *Sus domesticus* (2)	USA (10)	Experimentally infected pigs with local strains of the parasite (10)	[22]
Domesticated pigs, *Sus domesticus* (1–4)	Ecuador (6)	Quinidé and Súa Districts, Esmeraldas Province (6)	[21]
Domesticated pigs, *Sus domesticus*	China (1)		GenBank accession no: GU070737
Humans (12)	Uganda (17)	Villages ranged in south west of Kabale district (17)	[22]
Human (1)	China (2)	Unknown (2)	[58]
Humans (4)	Ecuador (12)	Quinidé and Súa Districts, Esmeraldas Province (12)	[21]
Human	China (6)	Zhangjiang, Guangdong Province.	GenBank accession nos: GU385218, AM993017-AM993021
Baboons, *Papiohamadryas* (5)	Denmark (25)	Copenhagen Zoo (12),Knuthenborg Park (13)	[58]
Baboons, *Papioanubis* (2) Baboon, *Papioanubis/P. cynocephalus* (1) Baboon, *Papioanubis/P. hamadryas* (1)	USA (9) USA (2) USA (1)	Southwest National Primate Research Center (SNPRC) Texas (12)	[58]
African Green Monkey, *Chlorocebussabaeus* (4)	Saint Kitts (12)	Feral population (11)	This Study

### Amplification of genetic markers and sequencing

Partial sequences of the two mitochondrial genes *rrn*L*nad*1 were obtained for all samples except for *Trichuris* from African green monkey, for which only the *rrn*L gene was sequenced. 562 bp of the *nad*1 gene was amplified using forward SuiND1_F (5′-CGAGCTTATATAGGTATTTCTCAACG-3′) and reverse SuiND1_R (5′-CGTTGTAGCCTCTTACTAATTCTCTTT-3′) primers while 422 bp of the *rrn*L gene was amplified using primers, forward TrirrnL_F (5′-TGTAAWTCTCCTGCCCAATGA) and reverse TrirrnL_R (5′-CGGTTTAAACTCAAATCACGTA). The PCR conditions were identical for both markers and were conducted in a total volume of 20 μl using 1 μl DNA as template. PCR ingredients were: 1X PCR buffer, 0.2 mM of each dNTP, 0.4 mM of each primer pair, 2.0 mM MgCl_2_, and 1 U of Hot Start DNA-polymerase (Ampliqon). PCR conditions were initial denaturation at 95 °C for 15 min followed by 35 cycles consisting of 95 °C for 30 s, 55 °C for 30 s and 72 °C for 1 min and a final extension at 72 °C for 10 min. Agarose gel electrophoresis (1.5 %) was used to verify amplification of a single fragment of the expected size. PCR products were enzymatically cleaned prior to sequencing using 10 μl of PCR product, 1 μl Exonuclease I and 2 μl Fast AP Thermosensitive Alkaline Phosphotase (1 U/μl) (Fermentas). The samples were incubated for 15 min at 37 °C followed by 15 min at 85 °C. Finally, cleaned amplicons were sequenced in both directions using same primers used for PCR by Macrogen Inc. in Seoul, South Korea.

### Genetic variation and phylogenetic relationships

Forward and reverse sequences from each sample were checked, edited manually and assembled using vector NTI [23] and then trimmed using BioEdit [24]. Genetic relatedness and evolutionary relationship were analysed for each of the two markers using 410 bp and 397 bp of the *nad*1 and *rrn*L genes, respectively. Two sequences of the *nad*1 and *rrn*L from the Chinese human and pig *Trichuris* mitochondrial genomes (Accession No: GU385218 and GU070737, respectively) were included in the dataset. Also, *T. trichiurarrn*L gene sequences from humans in China were included in the dataset (Accession no. AM993017-AM993021). Phylogenetic relationship was inferred using NJ and ML phylogenetic trees in MEGA v6.1 [25]. The best-to-fit substitution model was identified using jModelTest0.1.1 [26] under Akaike information criterion (AIC) [27]. *Trichinellaspiralis* was used as outgroup (Accession No: AF293969). The most parsimonious network was inferred by the Neighbor-net method using SplitsTree v.4.13.1 [28]. Neighbor-net network can reveal ambiguous and incompatible sites which usually appear as a reticulate structure in the network.

### Population genetic structure

Analysis of Molecular Variance (AMOVA) was used to estimate the Fixation index, F_st_ between *Trichuris* populations using Arlequin v.3.5.1.2 [29]. The F_st_ for the human and pig derived worms were calculated in separate analysis using the geographical origin of the worms to define the different populations. As the origin of *Trichuris* collected from baboons was unknown due to previous transport between zoological gardens, we used the phylogenetic analysis to define two populations for which F_st_ subsequently was estimated for. Lastly, F_st_ was estimated between the human and baboon worms that were found to cluster together. 10,000 permutations were used to test for differentiation between pairs of populations. The p-distance between the distinct clades identified in the phylogenetic analysis was calculated using MEGA v.6.1. [25].

### Demography, time of divergence and TMRCA

For uniparentally inherited DNA, the time to the most recent common ancestor (TMRCA) in generations is equal to the population size (N). The effective population sizes for the populations of *T. trichiura* and *T. suis* were calculated using the formula Θ = 2N_eff_μ where Θ (theta) is the genetic diversity of a population, μ is the mutation rate per gene and N_eff_ is the effective population size. Theta (Θ) and the ancestral history were estimated from Genetree [30] using a concatenated dataset of the two markers. First, sequences were aligned and imported to Map modules in the SNAP workbench [31] to collapse the sequences to haplotypes excluding sites which are indels and infinite site violations. Then, compatibility analysis using CladeEx revealed incompatible sites which were removed [31]. The simulations were repeated 5 times with 10 million runs with different random seeds to ensure convergence of the genealogies. The mutation rate of *Caenorhabditiselegans* was used which is 1.6 X 10^−7^ per site per generation [32] and found not to be significantly different from other free living nematodes [33]. To obtain the mutation rate per gene, the mutation rate per site was multiplied by the number of nucleotides used (807 nt for both markers) giving 1.29 X 10^−4^ per gene per generation.

BEAST v.1.6.1 [34] was used to infer the phylogeny and the divergence time using the Bayesian statistical framework and the concatenated dataset with *Trichuris* from pigs and humans as monophyletic groups. Different mutation models were used and the final analysis was done using strict molecular clock with a normal distributed substitution rate of 1.6 X 10^−7^(±0.3 X 10^−7^) based on the value of *C. elegans* [32]. The substitution model used here was Hasegawa-Kishino-Yano (HKY) with gamma distribution as best to fit model based on AIC [27] in jModelTest0.1.1 [26]. Yule prior, which is suitable for datasets that combine different species, was used as a tree prior with a random starting tree. Markov chain Monte Carlo (MCMC) chains were run for iterations with a burn in value of 1000. Tracer v.1.6 was used to analyse log files and to check whether the MCMC chains were sufficient by recording effective sample size values to be above 200, which was the case for all the parameters. The three log files of the three independent runs were combined using log combiner v1.6.1 [34]. Tree Annotater v1.6.1. [34] was used to summarize samples from the posterior on maximum credibility tree and the posterior probability limit set to 0.5. Figtree v1.3.1 [34] was used to depict the tree.

Divergence time was also estimated for the human and pig *Trichuris* populations using IMa2 based on an isolation and migration model [35] using the concatenated dataset. Priors used for these data sets were: for *T. trichiura*, t (the upper bound of splitting time) = 295, q (upper bound of population size) = 750, while for *T. suis* t = 40, q = 100. For both data sets, HKY substitution model was used, no migration between populations after splitting was assumed (m = 0), 20 Markov chains with geometric heating scheme (the first and second heating parameters were 0.96 and 0.90, respectively) and 10^6^ burn-in steps with 10^5^ sampling genealogies were used. Three independent runs were conducted with different seed numbers to assess the convergence.

## Results

### Phylogenetic analyses

**Fig. 1:**
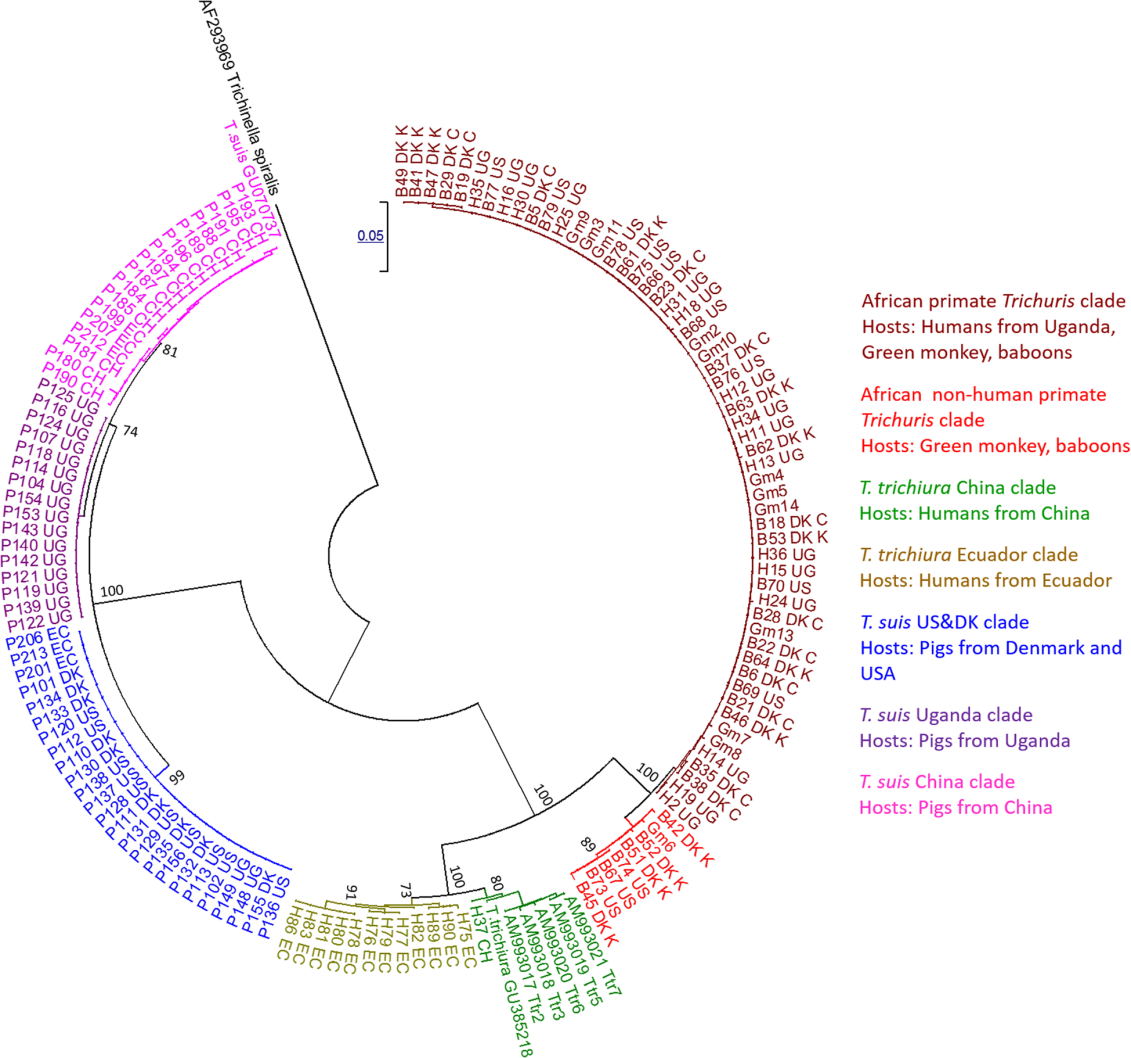
Phylogenetic relationship between different *Trichuris* populations inferred by Neighbor Joining (NJ) tree based on the *rrn*L gene and the Tamura-Nei with gamma distribution model. Seven major clades were identified and are indicated by different colors. *T. trichiura* from humans from Uganda clustered in one clade together with most *Trichuris* from baboons and African green monkey and are indicated by the maroon color (). Seven *Trichuris* from baboons and one from African green monkey clustered in a distinct clade and are indicated by the red color (). *T. trichiura* from China were distinct and are indicated by the green color () while worms from Ecuador are indicated by light green (). The other three clades include *Trichuris* from pigs. *T. suis* populations from USA and Denmark clustered together and are indicated by the blue color (), whereas *T. suis* from China and Uganda are indicated by pink () and purple (), respectively. Sample key are: B: Baboon, H: Human, P: Pigs, Gm: African green monkey; US, USA; Ch, China; UG, Uganda; DK, Denmark (C for Copenhagen Zoo and K for Knuthenborg)

We used two mitochondrial markers to infer the genetic relationships and the evolutionary history between different *Trichuris* populations obtained from humans, non-human primates and pigs from various geographical regions (Table 1). Partial sequences of the large ribosomal subunit (*rrn*L) and NADH dehydrogenase subunit 1 (*nad*1) were generated for all worms except for *Trichuris* from African green monkey, for which only the *rrn*L gene was sequenced (GenBank accession numbers: KU524489-KU524606 and KU524607-KU524730). The phylogenetic relationship was found to be identical when inferred using neighbor joining (NJ) and maximum likelihood (ML) clustering methods and for both genes, and hence only the NJ tree for the *rrn*L gene is shown in Fig. 1. For the ML tree, Hasegawa-Kishino-Yano with gamma distribution (HKY + G) model was used for the *nad*1 gene and Tamura-Nei with gamma distribution (TrN + G) model for the *rrn*L gene as the best-to-fit substitution models. It is noteworthy that two samples (two *T. trichiura* from humans, one in Uganda and one in China) showed double peaks in the chromatogram of the *nad*1 gene and gave conflicting signals for the *rrn*L gene (clustered in the pig clades). This may indicate co-amplification of nuclear mitochondrial pseudogenes (numts) or a heteroplasmy. Hence, these samples were excluded from further analyses.

**Fig. 2:**
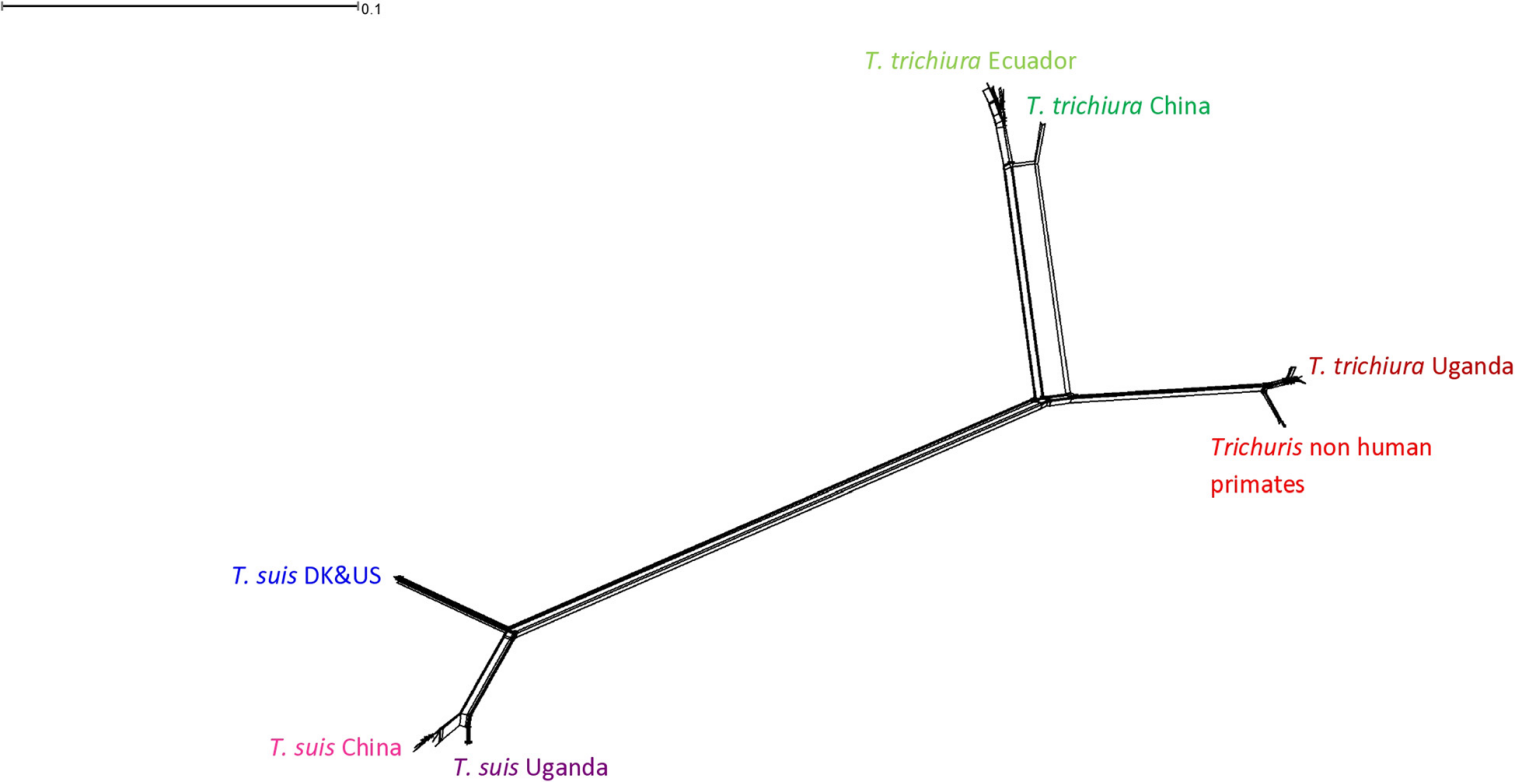
Neighbour-net network based on concatenated sequences of the *nad*1 and *rrn*L genes. The colors of the different populations are given in Fig. 1. *T. suis* from Ecuador cluster with worms from China, USA and Denmark and most *Trichuris* from non-human primates cluster with *T. trichiura* from Uganda

Phylogeographic distribution among *T. suis* populations was observed as worms from Uganda and from China were found in separate clades whereas *T. suis* from Denmark and USA clustered together. The Ecuadorian *T. suis* were found in two clades, namely the Denmark & USA clade (4 samples) and the China clade (3 samples). In addition, two pig worms from Uganda clustered with *T. suis* from Denmark & USA. Phylogeographic structure was also observed for worms recovered from humans as *T. trichiura* from Uganda, China and Ecuador were found in separate clades. The majority of the baboon and green monkey worms clustered with the human *T. trichiura* from Uganda. Additionally, a few baboon samples (*n* = 7) and a single green monkey worm grouped together in a different clade (*Trichuris* non-human primates). The Neighbor-net network identified splits that correspond to the clades in the NJ tree (Fig. 2).

### Population genetic structure (F_st_)

AMOVA was used to analyse the degree of genetic differentiation (F_st_) between *Trichuris* populations and is summarized in Tables 2 and 3 and is given for each of the two markers, but excluding *T. suis* from Ecuador as they cluster both with worms from China and Denmark. In general all the populations were highly differentiated as F_st_ values are above 0.25. *Trichuris* from baboons and green monkey in the two different clades (*Trichuris* African non-human primates and *Trichuris* African Primates) were highly differentiated with F_st_ values of 0.363 for *rrn*L (*P* < 0.01) and 0.471 for *nad*1 (*P* < 0.01). In contrast, human, baboon and green monkey *Trichuris* in the *T. trichiura* Afican clade represented undifferentiated populations (F_st_ < 0.05, *P* > 0.05). The genetic distances (p-distance) between and within each clade are given in Additional file 1: Table S1 for the *nad*1 and *rrn*L genes, respectively.

**Table 2:** Pairwise estimations of population differentiation (F_st_) between populations of *T. suis* and *T. trichiura* for the *nad*1 gene (below the diagonal) and the *rrn*L gene (above the diagonal). Level of significance is based on 10,000 permutations

	*T. suis* DK	*T. suis* Uganda	*T. suis* China	*T. suis* USA
*T. suis* DK		0. 981***	0.914***	0.000
*T. suis* Uganda	0.997***		0. 735***	0.981***
*T. suis* China	0.942***	0.876***		0. 914***
*T. suis* USA	0.016	0.995***	0.939***	

***P < 0.001

**Table 3:** Pairwise estimations of population differentiation (F_st_) between populations of *T. suis* and *T. trichiura* for the *nad*1 gene (below the diagonal) and the *rrn*L gene (above the diagonal). Level of significance is based on 10,000 permutations

	*T. trichiura* Uganda	*T. trichiura* China	*T. trichiura* Ecuador
*T. trichiura* Uganda		0.979**	0.932***
*T. trichiura* China	0.984**		0.551*
*T. trichiura* Ecuador	0.967***	0.778*	

*P < 0.05, **P < 0.01, ***P < 0.001

### TMRCA and the divergence time of the human and pig *Trichuris* populations

**Fig. 3:**
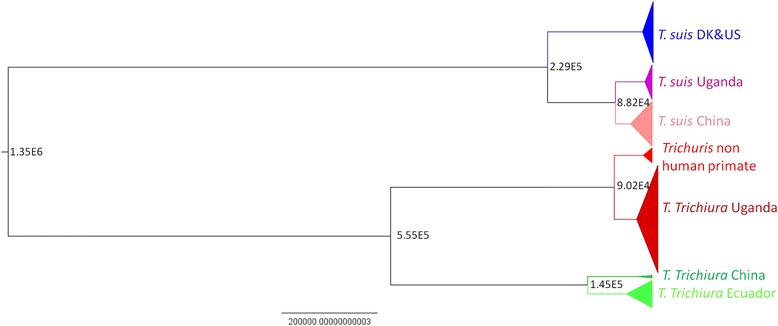
Bayesian phylogeny of the different primates and pig *Trichuris* populations. The different clades are indicated with the same colors used in the phylogenetic tree in Fig. 1. All nodes are supported by >99 % posterior support. Branch lengths are scaled in number of generations with the scale axis representing 200,000 generations. Median estimates of the divergence time are given at each node by number of generation

The estimated Θ was 20.7 ± 6.68 for *T. suis* and 146.49 ± 50.1 for *T. trichiura* from humans and the Genetree for their populations is provided in Additional file 2: Figure S1. Hence, the TMRCA in generations for *T. suis* equals 80,000 generations (upper estimate is 110,000 and lower estimate 60,000) and 560,000 generations for *T. trichiura* (upper estimate is 760,000 and lower estimate is 390,000). The time of divergence between the different populations as estimated by BEAST is given at each node in number of generations (Fig. 3) and by IMa2 in Additional file 3: Figure S2. BEAST, IMa2 and Genetree gave similar results for the time of divergence of the human *Trichuris* populations. However, the estimated time of divergence for *T. suis* populations was nearly three times older for BEAST and IMa2 than Genetree. Genetree relies on importance sampling (IS) algorithm for the coalescent simulations while BEAST and IMa2 each rely on correlated sampling (CS). As the IS algorithm is more suitable for data of low polymorphism [36], its estimate may be the most reliable in our case. There were two main divergence events in *T. suis* and *T. trichiura* populations. For *T. suis* there was an ancient split between the USA/Denmark populations and the China/Uganda populations (80,000-240,000 generations) and a more recent split between the populations from China and Uganda (32,000-90,000 generations). For *T. trichiura*, the first split is between the Uganda population and China/Ecuador populations (500,000 generations) and a more recent split between the China and Ecuador populations (120,000 generations).

## Discussion

**Fig. 4:**
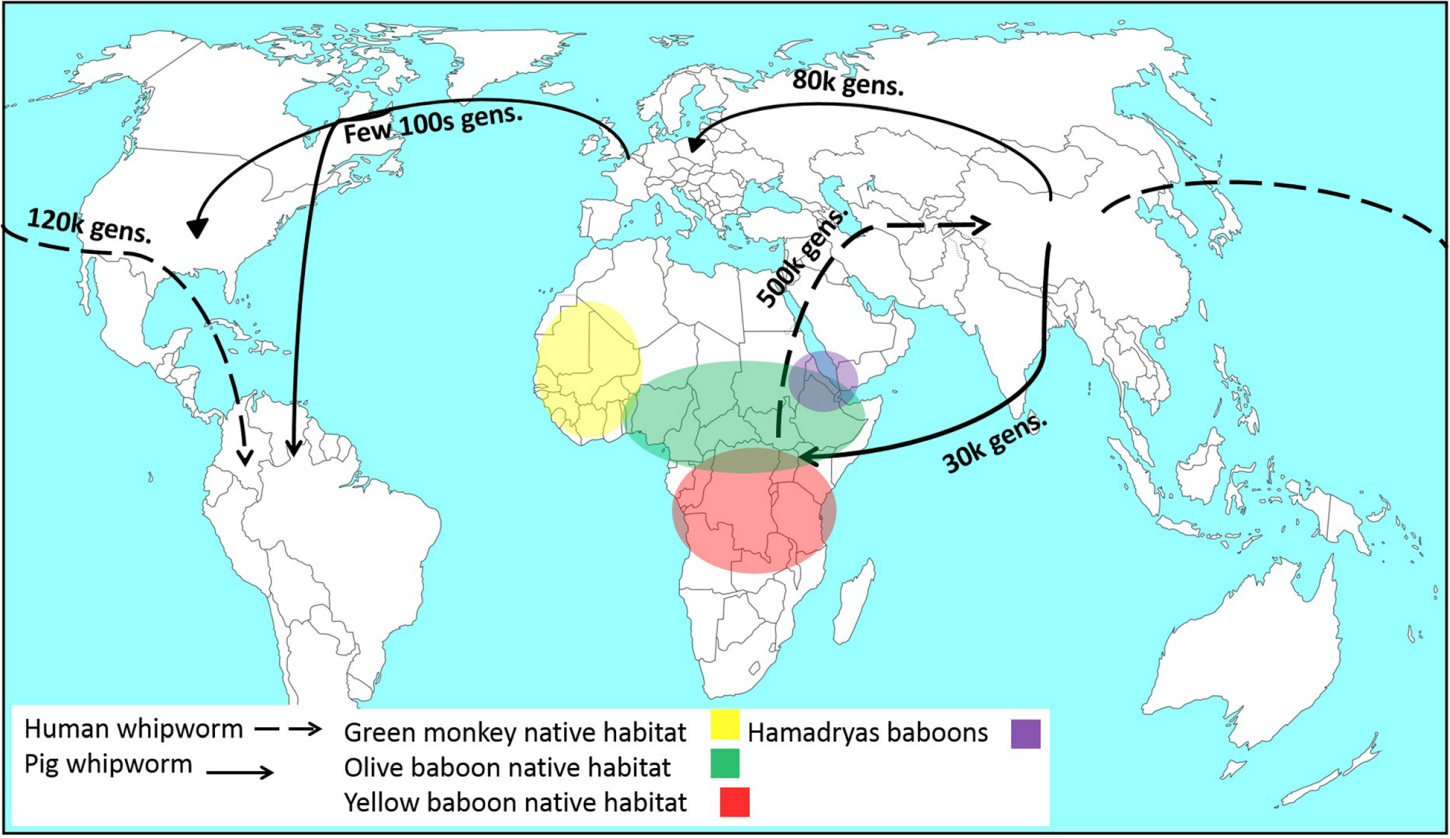
Summary of the evolutionary history showing possible dispersal routes of the human whipworms (dashed line) and pig whipworms (solid line) with the estimated time of divergence given as number of generations as estimated by Genetree. The native habitats in Africa of the different non-human primates (olive baboon (green), hamadrya baboons (purple), Yellow baboon (red) and African green monkey (yellow)) are indicated in the map. The origin of human *Trichuris* is believed to be in Africa where the parasite was transmitted to humans through early ancestors of primates while pigs evolved in China where it presumably acquired whipworms. Source of map: http://d-maps.com/carte.php?num_car=13180&lang=en. Map modified using Microsoft PowerPoint and GIMP 2

Herein, we investigated the evolutionary and genetic relationships between populations of *Trichuris* from primates and pigs from different geographical areas in order to infer the evolutionary and demographic history of the parasites, and to identify possible environmental and anthropological factors driving their spread across the globe. In this study *Trichuris* from humans and pigs were genetically very distinct with independent demographic histories as summarised in Fig. 4 and discussed below.

The coalescent analysis identified two divergence events in *T. suis* populations. First an ancient split between the DK/USA and the Chinese/Ugandan populations around 80,000 generations ago with a second more recent divergence between the China and Uganda populations 32,000 generations ago. Interestingly, this latter split between *T. suis* from China and Uganda is in line with that of their host [37]. Common alleles between the domesticated pigs of Far East and East African origin have been identified suggesting close evolutionary relationships between pigs in these regions [37, 38]. First, this may reflect transport of pigs from the Far East by the European trading routes to Africa a few hundred years ago. Secondly, domesticated pigs may have been introduced to Africa by trading between the ancient civilizations in Africa and Far East or the settlement of Austronesian peoples in East Africa nearly seven thousand years ago [37]. Given the high genetic differentiation between the *T. suis* populations of China and Uganda and assuming one to three generations for *T. suis* per year, the divergence between Chinese and Uganda *T. suis* population happened 32,000-10,700 years ago. Hence, it is unlikely that *T. suis* was introduced by an European intermediary only a few hundred years ago but may be traced back to the introduction of domesticated pigs from the Far East thousands of years ago. However, this does not exclude other waves of recent introduction of *T. suis* in domesticated pigs from the Far East. A recent study found that *A. lumbricoides* in humans on Zanzibar were closely related to worms from Bangladesh [7] suggesting parasite transportation between the Far East and the Indian subcontinent and East Africa.

Intriguingly, the *T. suis* population in Uganda was found to be monomorphic for both markers. This may relate to either a founder effect (the establishment of a new population from few individuals derived from a much larger population) or a selective sweep (strong positive natural selection of some few genotypes) or a combination of both factors. Such a selective sweep might have occurred due to new adaptations to host physiology in the new environment or, more likely, due to a recent bottleneck in the pig populations: e.g. African swine fever outbreaks in Uganda [39] resulted in subsequent reductions in molecular variation among the parasites [40]. However, due to our small sample size further worms should be analysed to confirm this hypothesis.

Several studies have reported high genetic distinctiveness between European and Chinese pigs [41, 42] in line with our observation for *T. suis* from Denmark and China. However, the divergence time between the pigs in China and Europe has been estimated to be roughly a million years ago [42] which is much older than the divergence time estimated for the Chinese and Danish *T. suis* populations in our study (80,000 years assuming one generation/year). In principle, when populations suffer from many bottlenecks, the coalescent will date back to the most recent bottleneck rather than the most recent common ancestor [5]. Considering the bottlenecks that the pig populations have been through during their migration and transport across Eurasia, with the most recent being nearly 20,000 years ago during the last glacial maxima [42], this may also have resulted in bottlenecks of the associated parasites. In addition, as *T. suis* can survive in the environment for at least 11 years [43] the number of generations per year is very hard to estimate and could be as low as 0.09 generations/year.

*T. suis* from Denmark and USA were found to cluster together (Fig. 1) with their populations being undifferentiated. This is consistent with other parasites of domestic pigs such as *Trichinellaspiralis*, which was introduced to the Americas by Europeans [44]. However, two *T. suis* from Uganda clustered with worms from Denmark and USA suggesting recent transport of pigs between these continents. The clustering of *T. suis* from Ecuador with the populations from China and DK/USA may reflect introgression between pigs from Europe and China during the industrial revolution in Europe in the 20th century as found in a previous study [21].

As for pig worms two divergence events were also observed for human derived *Trichuris*, one ancient (~500,000 generations) divergence between Uganda and China and a more recent one between China and Ecuador (~120,000 generations). Assuming the highest and lowest number of generations to be 1 and 3, respectively the divergence times were 500,000-160,000 generations and 120,000-40,000 generations for the Ugandan/Chinese and Chinese/Ecuadorian populations, respectively. The split between the China and Uganda populations preceded the modern human (*Homo sapiens*) migration out of Africa to South East Asia around 60,000-100,000 years ago [45, 46] and the human settlement in Latin America 14,000-15,000 years ago [47]. There are two possibilities for this discrepancy. Firstly, one of the early human ancestors (e.g. *H. erectus*) may have transmitted *T. trichuris* when migrating out of Africa [48] but this would not explain how the parasite was then introduced into Latin America. Secondly, the mutation rate of the free living nematode (*C. elegans*) may not be applicable to parasitic nematodes as the mutation rate normally is higher for the latter [49]. *T. trichiura* eggs in Brazil dating back to 6000–7000 BP [50] suggests that the parasite was introduced to the New World with the human migration much earlier than the Columbian colonization [51], which is concordant with our findings.

Green monkeys, olive, yellow and hamadryas baboons are indigenous species in central and western Africa and the introduction of the green monkey into Saint Kitts by the French in the late Seventeenth century [52] may explain why the majority of the baboon and green monkey worms clustered with human worms from Uganda. However, seven worms from baboons from both Denmark and USA and one from green monkey from St. Kitts were found in a separate clade (*Trichuris* African non-human primate), suggesting that different populations are circulating among these hosts’ species although they were sampled from the same habitat. Since many of these worms were sampled from unnatural habitats (zoos), the causes for this differentiation could not be investigated. However, in a sympatric natural transmission area in Uganda some *Trichuris* genotypes were found among all seven investigated non-human primates, whereas other genotypes seem to be more host specific [13]. Hence, in the past, *Trichuris* in primates in Africa may have been isolated either by geography or by host species leading to population differentiation followed by a more recent secondary sympatry [53]. As the *Trichuris* population between humans from Uganda and the non-human primates are undifferentiated this suggests continuous or recent gene flow between the host species and suggests Africa as the origin of *Trichuris* in primates.

Unlike whipworms, it is expected that host shift of the giant roundworm *Ascaris* in humans and pigs took place during animal domestication in the Neolithic period 10,000 years ago [7, 12]. This is supported by their very close genetic relationship [7, 54] and that *Ascaris* among non-human primates is observed rarely [55], suggesting that this parasite represents a ‘souvenir parasite’ [9]. Their different evolutionary history is interesting as *Trichuris* and *Ascaris* share several parasitic traits such as mode of transmission and infection and these two parasite species therefore usually co-occur today [56].

## Conclusions

We inferred the possible demographic history of *Trichuris* from humans and pigs and their potential evolutionary epicenter, namely in primates in Africa. We suggest that *Trichuris* was dispersed to Asia with human ancestors and that host switching to pigs occurred in China where pigs evolved [42]. *T. suis* was then spread across the globe mainly by anthropogenic factors. We found that *T. trichiura* in humans in Africa is genetically similar to *Trichuris* in non-human primates of African origin suggesting that *Trichuris* in humans represents a heirloom parasite. Further studies should investigate the genetic relationships of whipworms from different primates and other host species living in natural habitats in order to explore their demographic history and evolutionary origins and to identify the possible switching events between host species. Moreover, since we relied only on mitochondrial markers to resolve the evolutionary history of the parasite which in certain cases might be problematic due to the presence of the mitochondrial pseudogenes (numts), heteroplasmy or incomplete lineage sorting [57] further studies should also include suitable nuclear markers to confirm our findings.

## Additional files

Additional file 1: Table S1. Genetic distances within and between different clades identified by the phylogenetic analyses. See Figs. 1 and 2 for information on population definition. (DOC 41 kb) Additional file 2: Figure S1. The gene genealogy inferred by Genetree of (A) *T. suis* populations and (B) *T. trichiura* populations. Solid circles indicate mutations in the genealogy. (JPG 1237 kb) Additional file 3: Figure S2. Splitting time based on the isolation and migration model between (A) *T. suis* populations and (B) *T. trichiura* populations. The horizontal axis represents the number of generations since splitting which were estimated by dividing the splitting times between populations (t_0_ and t_1_) and time to most recent common ancestor (t_mrca_) by the mutation rate per gene per generation (μ) while the vertical axis is the posterior probability density. (JPG 149 kb)
