# Supplementary material for: Whipworms in humans and pigs: origins and demography
Source: Parasit Vectors. 2016 Jan 22;9:37. doi: 10.1186/s13071-016-1325-8 (PMC4724142; doi:10.1186/s13071-016-1325-8)
Supplement: Additional file 1: Table S1. — Genetic distances within and between different clades identified by the phylogenetic analyses. See Figs. 1 and 2 for information on population definition. (DOC 41 kb) [file 13071_2016_1325_MOESM1_ESM.doc]

Supplementary Table 1. Genetic distances within and between different clades identified by the phylogenetic analyses. See Figure 1 and 2 for information on population definition.

| ***nad*1 gene** | *T. trichiura* Uganda | *Trichuris* non-human primate | *T. trichiura* Ecuador | *T. trichiura* China | *T. suis* DK&USA | *T. suis* China | *T. suis* Uganda |
| --- | --- | --- | --- | --- | --- | --- | --- |
| *T. trichiura* Uganda | 0.4 |  |  |  |  |  |  |
| *Trichuris* non-human primate | 2.8 | 0.001 |  |  |  |  |  |
| *T. trichiura* Ecuador | 23 | 23.0 | 1.5 |  |  |  |  |
| *T. trichiura* China | 20.4 | 20.6 | 6.7 | 1.5 |  |  |  |
| *T. suis* DK & USA | 33.6 | 34.0 | 36.1 | 35.2 | 0.2 |  |  |
| *T. suis* China | 35.1 | 34.9 | 36.3 | 36.4 | 11.9 | 1.0 |  |
| *T. suis* Uganda | 33.4 | 33.1 | 35.9 | 36.1 | 11.4 | 4.0 | 0.00 |
| ***rrn*L gene** | *T. trichiura* Uganda | *Trichuris* non-human primate | *T. trichiura* Ecuador | *T. trichiura* China | *T. suis* DK&USA | *T. suis* China | *T. suis* Uganda |
| *T. trichiura* Uganda | 0.1 |  |  |  |  |  |  |
| *Trichuris* baboon | 1.9 | 0.5 |  |  |  |  |  |
| *T. trichiura* Ecuador | 8.7 | 9.0 | 0.7 |  |  |  |  |
| *T. trichiura* China | 8.9 | 9.2 | 2.0 | 0.3 |  |  |  |
| *T. suis* DK & USA | 20.3 | 20.5 | 21.5 | 21.4 | 0.00 |  |  |
| *T. suis* China | 20.2 | 20.3 | 21.3 | 21.2 | 2.5 | 0.4 |  |
| *T. suis* Uganda | 20.1 | 20.2 | 21.3 | 21.1 | 2.1 | 0.9 | 0.00 |
