# Supplementary figures and images for: Whipworms in humans and pigs: origins and demography
Source: Parasit Vectors. 2016 Jan 22;9:37. doi: 10.1186/s13071-016-1325-8 (PMC4724142; doi:10.1186/s13071-016-1325-8)

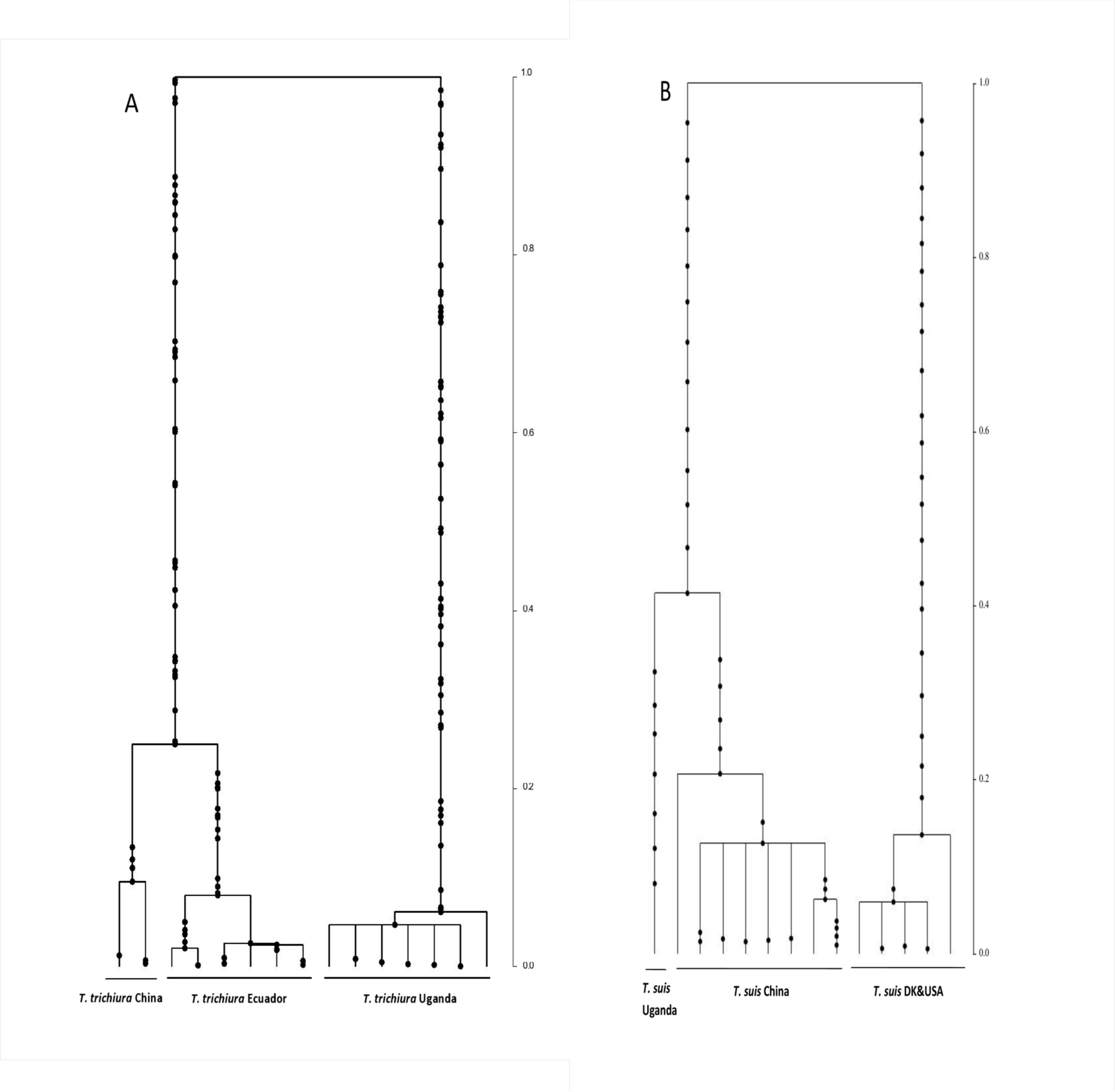

Supplement: Additional file 2: Figure S1. — The gene genealogy inferred by Genetree of (A) T. suis populations and (B) T. trichiura populations. Solid circles indicate mutations in the genealogy. (JPG 1237 kb) [file 13071_2016_1325_MOESM2_ESM.jpg]

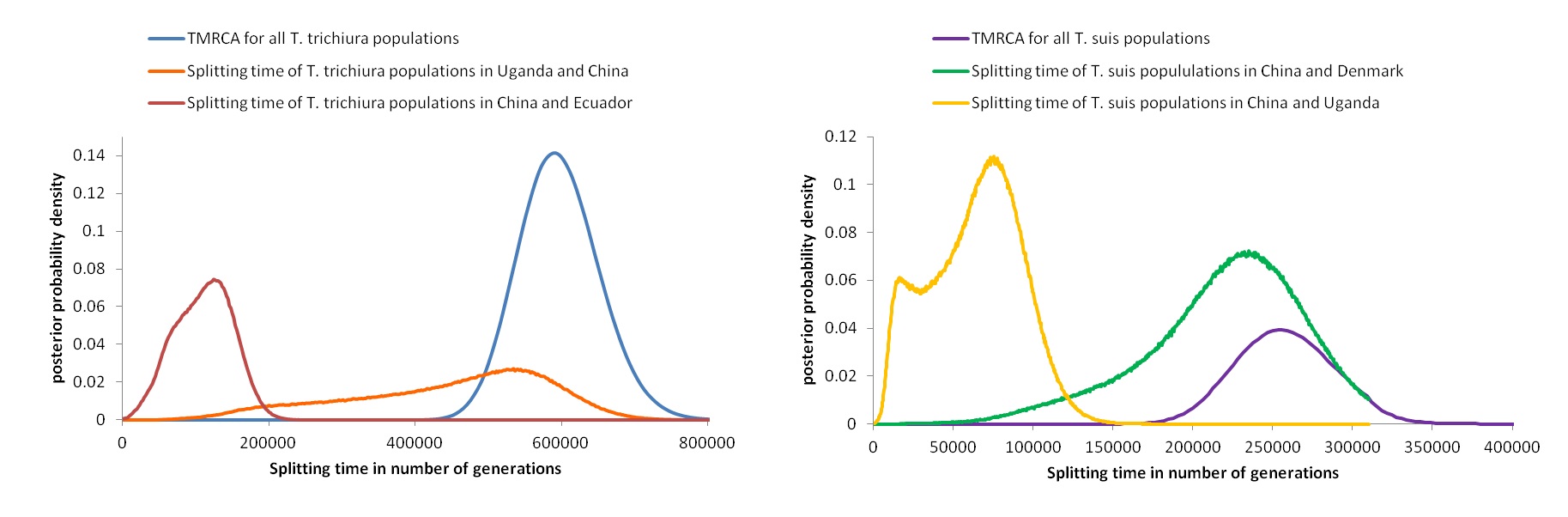

Supplement: Additional file 3: Figure S2. — Splitting time based on the isolation and migration model between (A) T. suis populations and (B) T. trichiura populations. The horizontal axis represents the number of generations since splitting which were estimated by dividing the splitting times between populations (t0 and t1) and time to most recent common ancestor (tmrca) by the mutation rate per gene per generation (μ) while the vertical axis is the posterior probability density. (JPG 149 kb) [file 13071_2016_1325_MOESM3_ESM.jpg]
